# Calculation and AFM Experimental Research on Slip Friction for Unlubricated Spherical Contact with Roughness Effect

**DOI:** 10.3390/mi12111428

**Published:** 2021-11-21

**Authors:** Shengguang Zhu, Liyong Ni

**Affiliations:** 1 University of Electronic Science and Technology of China, Zhongshan Institute, Zhongshan 528402, China; nily@zsc.edu.cn; 2 College of Mechanical Engineering, South China University of Technology, Guangzhou 510641, China

**Keywords:** sliding friction, potential barrier theory, rough surfaces in contact, micro/nano scale

## Abstract

Previous research on friction calculation models has mainly focused on static friction, whereas sliding friction calculation models are rarely reported. In this paper, a novel sliding friction model for realizing a dry spherical flat contact with a roughness effect at the micro/nano scale is proposed. This model yields the sliding friction by the change in the periodic substrate potential, adopts the basic assumptions of the Greenwood–Williamson random contact model about asperities, and assumes that the contact area between a rigid sphere and a nominal rough flat satisfies the condition of interfacial friction. It subsequently employs a statistical method to determine the total sliding friction force, and finally, the feasibility of this model presented is verified by atomic force microscopy friction experiments. The comparison results show that the deviations of the sliding friction force and coefficient between the theoretical calculated values and the experimental values are in a relatively acceptable range for the samples with a small plasticity index (Ψ≤1).

## 1. Introduction

Friction plays a very important role in engineering and daily life. Since Amontons formally proposed the two classical friction laws by experimental research in 1699, theoretical studies on friction have been conducted for hundreds of years, and many scientists have been actively exploring the origins of friction. Coulomb did not have the benefit of atomic-scale knowledge of surface morphology. In his search for a fundamental explanation of the origins of friction, he considered interlocking asperities and surface roughness as the causes [1]. In 1929, Tomlinson first proposed the molecular interaction theory of friction, according to which the cause of friction is the energy loss due to the intermolecular forces in the sliding process. With the gradual understanding of the phononic and electronic mechanisms, many energy dissipation friction models have been proposed, such as the Frenkel-Kontorova-Tomlinson model [2], Prandtl-Tomlinson model [3], Cobblestone Oscillator model [4], and so on.

A significant advancement was achieved in the 1950s when Bowden and Tabor [5] reported that when two surfaces touch each other, the actual microscopic area of the contact is typically 10,000 times less than the apparent macroscopic contact area. The vast majority of surfaces are not atomically flat, and when two such surfaces touch, their contact occurs only at their asperities. Consequently, friction is independent of the apparent contact area, whereas it is proportional to the true contact area. To understand friction, it is important to grasp the effects of surface morphology and load on the tribological performance. Hence, numerous models incorporate the results of the finite element method (FEM) [6,7,8] and sliding inception of a single asperity in a statistical representation of surface roughness [9] to obtain the maximum static friction. Typical models include the KE model [10], CKE model [11], and so on [12,13,14,15,16]. Based on Tabor’s friction theory, classical contact mechanics, finite element analysis, statistics, etc., many static friction calculation models for rough surface contact have been obtained, but they still lack sufficient experimental verification. However, the application of classical contact mechanics in the calculation of sliding friction is difficult, and the calculation models of sliding friction are rarely reported, particularly for rough surface contact, whereas it is easy to measure the sliding friction using modern techniques, such as quartz crystal microbalance, atomic force microscope (AFM) [17], etc. Sliding friction generally involves a stick-slip phenomenon, which was confirmed by Mate et al. [18], whose experimental results showed that the friction force on a probe tip fluctuates periodically with the position of a graphite sample. The change period was approximately 0.25 nm, which is identical to the honeycomb hexagonal structure of the graphite surface along the moving direction. The experiments of Mate et al. presented the relationship between the interfacial friction and the material microstructure for the first time. Therefore, the magnitude of sliding friction can be reflected by the change in the periodic substrate potential. However, the difficulty in the calculation of sliding friction is that the friction process is accompanied by energy dissipation, wear, etc. The relevant models [19] are basically in the stage of numerical simulation and have not been verified by experiments.

Based on the discussion above, a novel calculation model of unlubricated sliding friction with a roughness effect was investigated in this study. We established an unlubricated spherical contact with the basic assumptions of the Greenwood-Williamson random contact model and assumed that the real contact area satisfies the interfacial friction condition. Subsequently, we used the contact interfacial potential theory and a statistical method to solve the calculation problem of sliding friction. Finally, the feasibility of the model proposed in this paper was verified by AFM friction experiments.

## 2. Modeling

### 2.1. Contact Model of Sphere and Nominal Rough Flat

**Figure 1:**
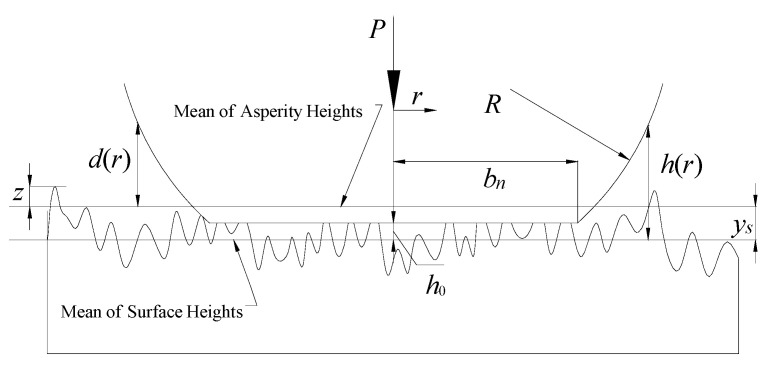
Contact model of rigid sphere and nominally rough flat [22].

As shown in [9,20], the roughness of a sphere can be transferred to a nominal flat without changing the original contact problem; hence, a model of an unlubricated contact between a smooth sphere and a nominal rough flat is presented in Figure 1. The sphere and the contacting asperities deform, resulting in the real contact area, $A_{0}$, which supports the normal load, *P*. The peak of the sphere is flattened by the displacement, ω, forming a circular contact area of radius, $b_{n}$. It should be stated that representing the rough circular contact area as a flat is only a simplified approximation. *R* is the radius of the rigid sphere, and the separations, d(r) and h(r), denote the distance from the sphere surface to the mean of the asperity summit heights and the mean of the surface heights at the radial coordinate, *r*, respectively [21].

Moreover, the following basic assumptions used in [9,20] for a nominally flat and sphere contact are adopted in this study:

1. The rough surface is isotropic.
2. All asperity summits are spherical and have the same radius, ρ, whereas their heights vary randomly.
3. There is no interaction and bulk deformation of the asperities.

The contact friction between the smooth ball and the rough flat is a statistical result of the contact between each equivalent rough peak and the smooth rigid surface under the combined action of the normal and tangential loads. Therefore, the friction between a single rough peak and the smooth rigid surface is first analyzed.

### 2.2. Friction Model of Single Asperity Based on Potential Barrier Theory

**Figure 2:**
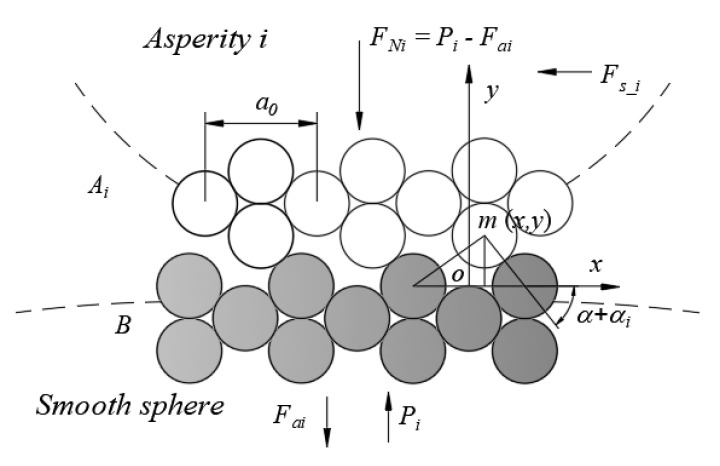
Schematic of contact between asperity *i* and smooth sphere.

Under the action of the normal force, a rough peak contacts the rigid smooth sphere surface and deforms. It is assumed that the positions of the interfacial atoms are fully adjusted during the deformation process, and no wear is caused by a macro slip. Simultaneously, it is assumed that the contact surface is smooth and clean, and there is no oxide and adsorption film formation. Therefore, the interfacial atoms of the two contact surfaces directly participate in the contact, and the contact parts meet the interfacial friction conditions [1]. Since a solid has strong volume dependence characteristics, the atoms on the interface can be regarded as dense stacked rigid balls, and because the diameter of the smooth sphere is much larger than that of a rough peak, the smooth sphere is approximately planar at the contact part. The contact of asperity *i* with the smooth sphere is shown in Figure 2.

According to the contact interfacial potential barrier theory [23], the position of an interfacial atom corresponds to a certain potential energy; to make the object move, the external force must overcome this interfacial atomic potential energy. The slip friction, $F_{\mathrm{slip}\_i}$, of asperity *i* is calculated as follows:

(1) $$F_{\mathrm{slip}\_i}=k_{i}\times g(T)\times \frac{\Delta U_{i}}{\Delta x_{i}}$$

where $k_{i}$ is the position commensurate coefficient, and if the atoms at the contact interface of asperity *i* are fully adjusted, then coefficient $k_{i}$ is defined as 1. g(T) is the temperature coefficient, which is extremely complicated to fix, because the friction process is accompanied by thermoelastic effect [24,25], environmental factor, and so on; an approach to obtain the temperature coefficient is by molecular dynamics calculations [15]. $\Delta U_{i}$ is the change in the interfacial potential energy of asperity *i* with displacement $\Delta x_{i}$, and it is given by [26] as follows:

(2) $$\Delta U_{i}=\bar{A_{i}}\times \Delta E\times \left[1-E^{*}(a^{*})\right]$$

where $\bar{A_{i}}$ is the real contact area of asperity *i*, ΔE is the adhesion energy per unit area at balance distance, $a_{m}$, and ΔE=w, where w is the potential energy or adhesion energy of the contact interface per unit area as follows [15]:

(3) $$w=\gamma_{A}+\gamma_{B}-\gamma_{\mathrm{AB}}$$

where $\gamma_{A}$ and $\gamma_{B}$ are the surface free energies of the two contact surfaces, respectively, and $\gamma_{\mathrm{AB}}$ is the interfacial free energy. For the same friction pair material, $\gamma_{A}=\gamma_{B}=\gamma$ and $\gamma_{\mathrm{AB}}\approx 0$, and thus, ΔE=2γ.

Moreover, $E^{*}(a^{*})$ is a universal approximate function expressed by the Rydberg function [26] as follows:

(4) $$E^{*}(a^{*})=\left(1+a^{*}\right)e^{-a^{*}}$$

where $a^{*}$ is the interfacial clearance after a proportional adjustment,

(5) $$a^{*}=(y-a_{m})/l_{s}$$

where *y* is the interfacial clearance, and $l_{s}$ is the length ratio parameter.

(6) $$l_{s}=\sqrt{2\gamma d/E_{\mathrm{int}}}\approx 2\sqrt{2\gamma /(12\pi E_{\mathrm{bod}}r_{\mathrm{WS}})}$$

where $E_{\mathrm{int}}$ is the elastic modulus of the interface, $E_{\mathrm{bod}}$ is the bulk modulus of elasticity, and $r_{\mathrm{WS}}$ is the Wigner-Seitz radius.

**Figure 3:**
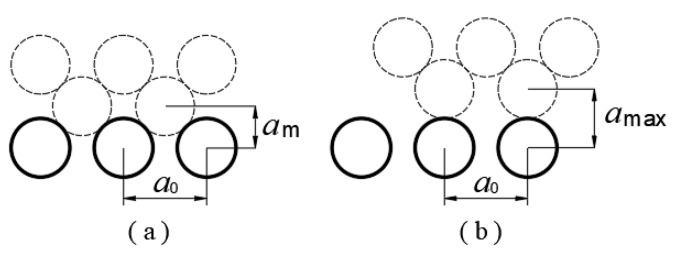
Diagram for calculation of variation in interfacial clearance. (**a**) Minimum clearance and (**b**) maximum clearance of FCC metals.

The $a^{*}$ in Equation (5) can be calculated based on the crystal structure [16]. For simplicity, only one type of friction material is analyzed in the present study. For a material with a face-centered cubic structure (FCC), as shown in Figure 3a,b, the variation in the interfacial clearance in each potential cycle can be obtained from

(7) $$\delta =a_{\max}-a_{m}=\frac{\sqrt{2}}{2}a_{0}-\frac{1}{2}a_{0}\approx 0.207a_{0}$$

where $a_{0}$ is the lattice constant. For a material with a body-centered cubic structure (BCC), the calculated result is the same ($\delta \approx 0.207a_{0}$). Therefore, for FCC and BCC materials, we obtain:

(8) $$a^{*}=\delta /l_{s}\approx 0.207a_{0}/l_{s}.$$

Substituting Equations (2), (4), and (8) into Equation (1), and using ΔE=2γ, the slip friction of a single rough peak of the same friction pair materials (FCC or BCC) in a single potential energy cycle can be obtained as follows:

(9) $$F_{\mathrm{slip}\_i}=\frac{k_{i}\times g(T)\times 2\gamma \times \bar{A_{i}}}{a_{0}}\left[1-\left(1+\frac{0.207a_{0}}{l_{s}}\right)e^{-\frac{0.207a_{0}}{l_{s}}}\right].$$

### 2.3. Slip Friction of Rough Surface Contact

The slip contact friction of rough surfaces is a statistical result of the friction of each asperity; therefore, using Equation (9), we have the polynomial form [27]:

(10) $$F_{\mathrm{slip}}=\sum_{i=1}^{n}F_{\mathrm{slip}\text{\_}i}=g(T)\frac{2\gamma }{a_{0}}\left[1-\left(1+\frac{0.207a_{0}}{l_{s}}\right)e^{-\frac{0.207a_{0}}{l_{s}}}\right]\sum_{i=1}^{n}k_{i}\cdot \bar{A_{i}}.$$

During loading, the real contact area, $\bar{A}$, of each individual asperity depends only on its own interference, ω, which is defined as ω=z−d. The total real contact area, $A_{0}$, can be obtained by summing the contributions of all contacting individual asperities using a statistical model [22], hence,

(11) $$A_{0}=\eta \int_{0}^{R}2\pi \left[\int_{d}^{\infty }\bar{A}\left(z-d\right)\phi \left(z\right)dz\right]rdr$$

where η is the area density of asperities, z is an asperity height (see Figure 1), and ϕ(z) is the statistical height distribution function of the asperities. The peak height distributions for most engineering surfaces are normal, and their Gaussian distribution is as follows:

(12) $$\phi (z)=\frac{1}{\sqrt{2\pi }\sigma_{s}}\exp(-0.5{\left(\frac{z}{\sigma_{s}}\right)}^{2})$$

where $\sigma_{s}$ is the standard deviation of the asperity summit heights. If the rough surface is non-Gaussian, the Pearson distribution can be used for the fitting [28].

The real area of a spherical contact, $\bar{A}$, has been studied by many scholars [6,29], and the calculation of the spherical contact area, $\bar{A}$, under a perfect slip condition is adopted from [29].

The critical interference, $\omega_{c}$, and the critical load, ${\bar{P}}_{c}$, in a perfect slip condition were provided by Brizmer et al. [29] as follows:

(13) $$\omega_{c}={\left(C_{v}\frac{\pi \left(1-v^{2}\right)}{2}\left(\frac{Y_{0}}{E}\right)\right)}^{2}\rho$$

(14) $${\bar{P}}_{c}=\frac{\pi^{3}Y_{0}}{6}C_{v}^{3}{\left(\rho \left(1-v^{2}\right)\left(\frac{Y_{0}}{E}\right)\right)}^{2}$$

where $C_{v}$ is the linear function of Poisson’s ratio, v (see Table 1). Parameters $Y_{0}$, E, and ρ are the virgin yield stress, Young modulus, and asperity summit radius, respectively.

Corresponding to the yield inception under the slip contact condition, the critical value of the contact area follows from the Hertz solution as

(15) $${\bar{A}}_{c}=\pi \omega_{c}\rho .$$

The results of the contact area and contact load during the slip as function of interference, ω, in the elastic regime, $\omega /\omega_{c}<1$, from the Hertz solution are

(16) $${\bar{A}}_{el}=\pi \omega \rho$$

(17) $${\bar{P}}_{el}=\frac{4}{3}E\rho^{1/2}\omega^{3/2}.$$

In the elastic-plastic regime, $\omega /\omega_{c}>1$, and the contact area and contact load during the slip from [29] are

(18) $${\bar{A}}_{ep}=\frac{\omega }{\omega_{c}}\left(1+\exp{\left(1-{\left(\frac{\omega }{\omega_{c}}\right)}^{\alpha }\right)}^{-1}\right){\bar{A}}_{c}$$

(19) $${\bar{P}}_{ep}={\left(\frac{\omega }{\omega_{c}}\right)}^{3/2}\left(1-\exp{\left(1-{\left(\frac{\omega }{\omega_{c}}\right)}^{\beta }\right)}^{-1}\right){\bar{P}}_{c}$$

where α and β are linear functions of Poisson’s ratio, v (see Table 1).

The total real contact area mainly divides into two parts (the area of elastic regime, $\omega /\omega_{c}<1$, and the area of elastic-plastic regime, $\omega /\omega_{c}>1$), so, substituting Equations (16) and (18) into Equation (11), one can obtain the total real contact area:

(20) $$A_{0}=2\pi^{2}\eta \rho \int_{0}^{R}\left[\int_{d}^{d+\omega_{c}}\left(z-d\right)\phi \left(z\right)dz+\int_{d+\omega_{c}}^{\infty }\left(z-d\right)(1+I^{\lambda })\phi \left(z\right)dz\right]rdr.$$

Moreover, using Equations (14), (17), and (19), the total contact load under the perfect slip condition is obtained statistically as follows [22]:

(21) $$P=2\pi \eta \int_{0}^{R}r\left[\frac{4}{3}E\rho^{1/2}\int_{d}^{d+\omega_{c}}{\left(z-d\right)}^{3/2}\phi (z)dz+\int_{d+\omega_{c}}^{\infty }{\left(\frac{z-d}{\omega_{c}}\right)}^{3/2}\left(1-I^{\kappa }\right){\bar{P}}_{c}\phi (z)dz\right]dr$$

where $I^{\lambda }$ in Equation (20) and $I^{\kappa }$ in Equation (21) have the following forms:

(22) $$I^{\lambda }=\exp{\left(1-{\left(\frac{\omega }{\omega_{c}}\right)}^{\alpha }\right)}^{-1}$$

(23) $$I^{\kappa }=\exp{\left(1-{\left(\frac{\omega }{\omega_{c}}\right)}^{\beta }\right)}^{-1}.$$

For the conciseness of the expression in Equation (10), we substituted this operator with symbol *D* as follows:

(24) $$D=1-\left(1+\frac{0.207a_{0}}{l_{s}}\right)e^{-\frac{0.207a_{0}}{l_{s}}}.$$

To simplify the calculation, assuming that the atoms at the contact interfaces of all asperities are fully adjusted, $k_{i}=1$, and using Equations (20) and (24), Equation (10) can be expressed in an integral form as follows:

(25) $$F_{\mathrm{slip}}=\frac{4\pi^{2}\eta \rho g(T)D\gamma }{a_{0}}\int_{0}^{R}\left[\int_{d}^{d+\omega_{c}}\left(z-d\right)\phi \left(z\right)dz+\int_{d+\omega_{c}}^{\infty }\left(z-d\right)(1+I^{\lambda })\phi \left(z\right)dz\right]rdr.$$

Regardless of the adhesion force, the slip friction coefficient can be simply expressed as

(26) $$\mu =\frac{F_{\mathrm{slip}}}{P}.$$

## 3. Experiments

To verify the feasibility of the slip friction calculation model established in this study, we prepare some rough flat samples and obtain their topography by AFM. Subsequently, AFM friction experiments are conducted on the samples using a spherical probe, and finally, the friction results obtained from the AFM experiments are compared with the friction results calculated theoretically. The specific experimental steps are as follows:

1. Sample preparation

The experiments examine the spherical contact friction for the same friction pair materials; therefore, the material of the ball probe is required to be consistent with that of the rough surface sample. The material of the mainstream AFM probe available in the market is monocrystalline silicon; therefore, a common silicon probe and the monocrystalline silicon wafer are preferred for probes and samples in this study. Si is the dominant material in MEMS, due to its crystal structure: the entire solid is made up of atoms in an orderly array [30].

There are many methods for producing surfaces with different roughness on a silicon wafer: micromachining, LIGA process, wet etching, and dry etching. In the experiments of this study, wet etching is conducted on an N-type polished silicon wafer to obtain samples. There are many reagents for wet etching, including acid etchants, alkaline corrosion agents, and organic corrosion agents. The corrosion solution used in the experiments is a mixture of potassium hydroxide solution (KOH + H_2_O, the mass fraction of KOH is 10%) and isopropanol solution (the mass fraction of isopropanol is 25%), and the ratio of the KOH solution to the isopropyl alcohol solution is 9:1. The corrosion mechanism is as follows [31]:

(27) $${\mathrm{KOH}+H}_{2}O\to K^{+}+2{\mathrm{OH}}^{-}+H^{+}$$

(28) $$\mathrm{Si}+2{\mathrm{OH}}^{-}+4H_{2}O\to {\mathrm{Si}(\mathrm{OH})}_{6}^{2-}+2H_{2}↑$$

(29) $${\mathrm{Si}(\mathrm{OH})}_{6}^{2-}{+6(\mathrm{CH}}_{3}{)}_{2}\mathrm{CHOH}\to {\left[{\mathrm{Si}(\mathrm{OC}}_{3}H_{7}{)}_{6}\right]}^{2-}+6H_{2}O.$$

In the experiments, samples 1–4 were obtained by placing [100] single polished monocrystalline silicon wafer (Guangzhou Fangdao Silicon Material Co., Ltd., Guangzhou, China) in the corrosion solution and etching at a constant temperature of 70 °C for 5 min, 10 min, 15 min, and 20 min, respectively. Samples with different roughness levels were prepared by different wet etching times. The sample size during corrosion is required to produce four subsamples, which not only ensures the consistency of the characteristics of the subsamples but also avoids the failure of the subsequent experiments due to a sole sample.

2. Sample topography parameter measurement

**Figure 4:**
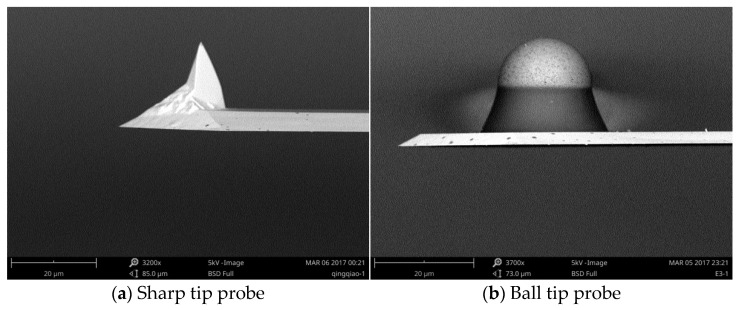
SEM images of probe tips: (**a**) sharp tip and (**b**) ball tip.

The topography and slip friction were measured via AFM (Beijing Nano-Instruments CSPM-4000, Guangzhou, China) using a beam deflection type equipment. For topography measurement, AFM can be conducted in two modes: contact and tap modes. In the process of topography scanning with the contact mode, the existence of friction makes the wear between the sample and the probe inevitable. In the tapping mode, because there is no friction and wear, the topography measurement accuracy is higher than that in the contact mode; however, a disadvantage of the tapping mode is that the scanning frequency is lower than that of the contact mode. In the experiments, although both modes can be selected for morphology sampling, the tap mode is preferred using a sharp probe (Tap190Al-G Budget Sensors, 190 kHz, 48 N/m) in this work. Generally, a sharp probe with a small elastic coefficient (<1 Nm^−1^) can be selected to reduce the normal load between the probe and the sample, as shown in Figure 4a. Regarding the structure and working principle of AFM, please refer to [32].

A high symmetry of rough peak height and density distributions is appropriate, because the established model of this study is based on the isotropy assumption of the Greenwood-Williamson random contact model. Before the topography scanning, the samples were ultrasonically cleaned in an alcohol solution for 15 min and subsequently ultrasonically cleaned in distilled water for 15 min. The microscope was operated under ambient conditions (temperature 20 ± 1 °C, relative humidity 70 ± 3%).

3. Surface energy measurement

A key parameter of the friction calculation model established based on the interfacial potential energy theory is the surface energy of the contact surface. Many studies provide the experimental and theoretical values of the surface free energies of several different materials; however, the surface energies of the samples in the present experiments may be different owing to the difference in the roughness. Therefore, the surface energy of each sample was measured in the experiments. The surface energy is calculated using the Owens-Wendt-Kaelble method [33]:

(30) $$\gamma_{L}\left(1+\cos\theta \right)=2\left(\sqrt{\gamma_{S}^{d}\gamma_{L}^{d}}+\sqrt{\gamma_{S}^{p}\gamma_{L}^{p}}\right)$$

where $\gamma_{L}$ is the surface tension of the liquid, θ is the contact angle, $\gamma_{S}^{d}$ and $\gamma_{S}^{p}$ are the dispersion and polar components of the surface energy of the solid, respectively, $\gamma_{L}^{d}$ and $\gamma_{L}^{p}$ are the dispersion and polar components of the surface tension of the liquid, respectively, and the surface energy of the solid, $\gamma_{S}$, is as follows:

(31) $$\gamma_{S}=\gamma_{S}^{d}+\gamma_{S}^{p}.$$

Therefore, using the parameters ($\gamma_{L}$, $\gamma_{L}^{d}$, and $\gamma_{L}^{p}$) of two test liquids and the contact angles with the measured samples, $\gamma_{S}^{d}$ and $\gamma_{S}^{p}$ can be calculated by Equation (30); finally, the free energy, $\gamma_{S}$, of each sample surface is obtained through Equation (31).

An SCA20 contact angle measuring instrument is used for measuring the contact angles in the experiments. Moreover, common distilled water and diiodomethane were used as test liquids; their surface tension values are listed in Table 2. The static contact angles were measured using the static drop method at room temperature. The contact angle of each sample was measured four times, and subsequently, the average value was taken. Substituting the values of Table 2 and measured angles of Table 3 into Equation (30), one can obtain the surface energy components of samples, finally, using Equation (31) to get the surface energy values of samples. The measurement and calculation results are summarized in Table 3.

4. Friction experiment

The main objective of this experiment is to verify the calculation models of sliding friction. For each sample, the friction loop curves are tested by changing the normal load to obtain the friction experimental values of each sample under different positions, and finally, the experimental values are compared with the theoretical calculated values.

Environmental factors, such as vibration, humidity, and wind force, significantly impact nanoscale measurements. To reduce the influence of environmental factors, the friction measurements were conducted in a glove box (Etelux Lab2000, Etelux Inert Gas System Company Limited, Beijing, China). The glove box is circularly filled with filtered nitrogen, and the water and oxygen contents are less than 0.1 ppm. However, the samples, similar to particularly insulators, are prone to static electricity in the glove box filled with high-purity dry nitrogen. Therefore, to minimize the static electricity force, an electrostatic removal device (SY-504 ionic copper rod, Shenzhen Shengyuan Anti-static Technology Co., Ltd., Shenzhen, China) is used in the experiments.

In the friction measurement, the friction loop curve function module of AFM and a spherical probe (see Figure 4b) are adopted. It can be seen from the scanning electron microscopy (SEM) image that the spherical tip surface is very smooth and can ideally simulate a smooth ball. The basic structure of the probe is composed of a substrate, cantilever, and needle tip. The commonly used materials are silicon and silicon nitride. Micro cantilevers generally have two shapes: triangular and rectangular. In the present experiments, a rectangular cantilever probe is adopted because it is more stable and sensitive to the transverse force than a triangular probe.

The elastic parameters provided by the manufacturer are typically the average values of the same batch of probes; however, in practical use, a probe needs to be accurately calibrated. The calibration method is generally to measure the width, thickness, length, and tip height (*w*, *b*, *l*, and *h*) of a probe using an optical or electron microscope. The normal force constant, $k_{n}$, and the transverse force constant, $k_{l}$, of the probe are calculated respectively as

(32) $$k_{n}=\frac{Ewb^{3}}{4l^{3}}$$

(33) $$k_{l}=\frac{Gwb^{3}}{3h^{2}l}$$

where *E* and *G* are the elastic modulus and shear modulus of the probe cantilever, respectively, whose values are provided by their manufacturer. In these experiments, a silicon probe with radius 9.55 µm was used to test the friction of the samples. SEM was conducted to measure the geometric parameters of the spherical probe (*w*, *b*, *l*, and *h*). The parameters of the spherical probe and the force constants calculated by Equations (32) and (33) are listed in Table 4.

The calculation formulas of normal contact force $F_{N}$ and transverse force $F_{L}$ in AFM are as follows:

(34) $$F_{N}=k_{n}S_{z}V_{N}$$

(35) $$F_{L}=\frac{3h}{2l}k_{l}S_{z}V_{L}$$

where *S_Z_* is the sensitivity and *V_N_* and *V_L_* are the normal voltage difference and horizontal voltage difference of the AFM photodetector, respectively.

**Figure 5:**
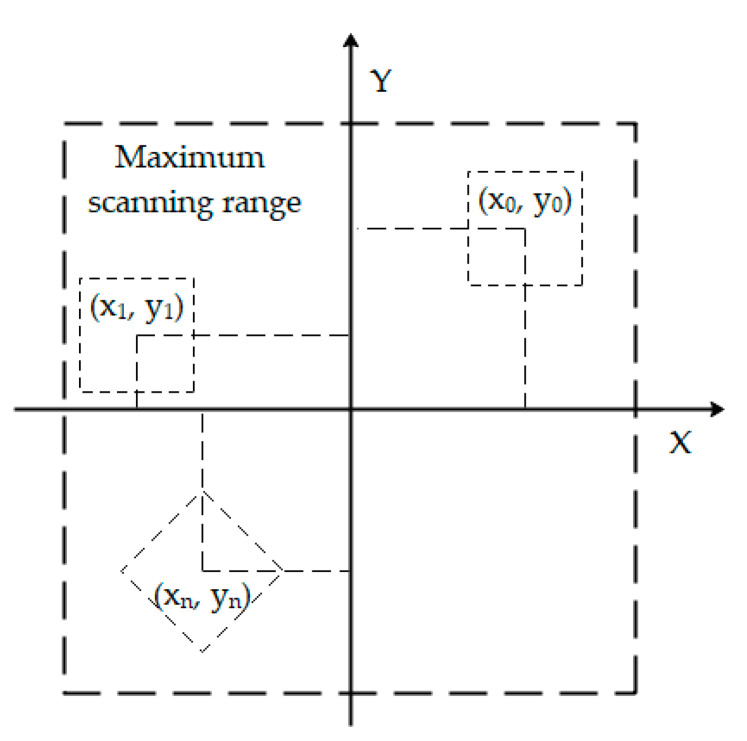
Scanning location, range, and angle setting by CSPM console.

Considering the wear effect of a probe on sample topography and to obtain a highly realistic statistical value of the influence of the topography parameters on friction, in the experiments, different positions must be changed after each friction loop scan. To reduce operations, such as needle withdrawal, moving sample position, and needle insertion, in the present experiments, the function of changing the scanning position, range, and angle within the maximum scanning range of the scanner provided by AFM control software (CSPM console) is used, as shown in Figure 5.

The maximum reference voltage of CSPM4000 is 1.99 V, and the maximum scanning range of scanner S8095 is 85,525 nm. The sampling length of the friction loop curve is 15 µm, and the resolution is set as 1024. Increment in the normal load is realized by increasing the reference voltage in AFM by a gradient. The voltage gradient of the ball probe is 0.2 V, ranging from 0.1 to 1.7 V. For each sample and each reference voltage, the friction loop curves are tested in three different positions by setting the scanning offset coordinates. For the speed, the minimum scanning frequency of 0.1 Hz is adopted.

## 4. Results and Discussion

### 4.1. Sample Surface Characterization

The surface topography scanning was performed twice for each sample by AMF; then, the topography images were analyzed by the NanoScope_Analysis (an image analysis software). Through the surface roughness analysis function of NanoScope_Analysis, we can get the mean values of the main surface roughness parameters (Sa, Sq, Ssk, Sku), which are summarized in Table 5. The value before the plus–minus sign is the average of the two sampling values for the same sample, and the number after the plus–minus sign is half of the difference between the two sampling values. The AFM operation of this experiment is strictly in accordance with the user manual. The ratio of difference to mean in Table 5 is less than 7%, indicating that there is no uncertainty in the sampling process.

**Figure 6:**
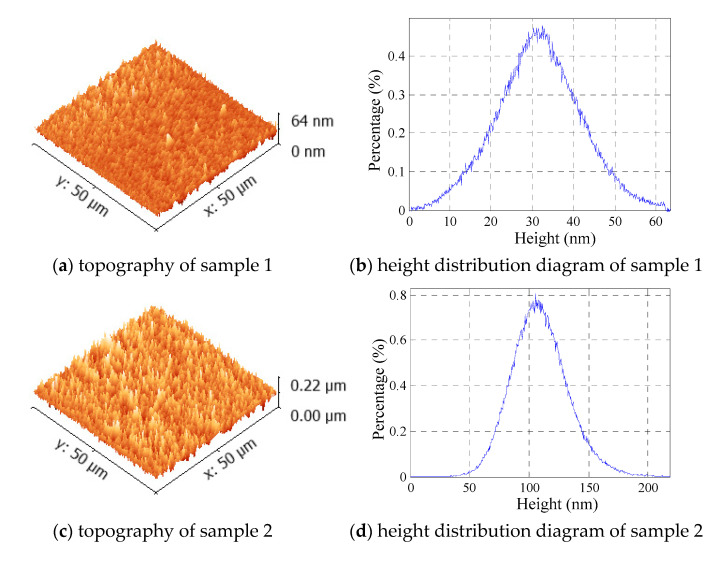

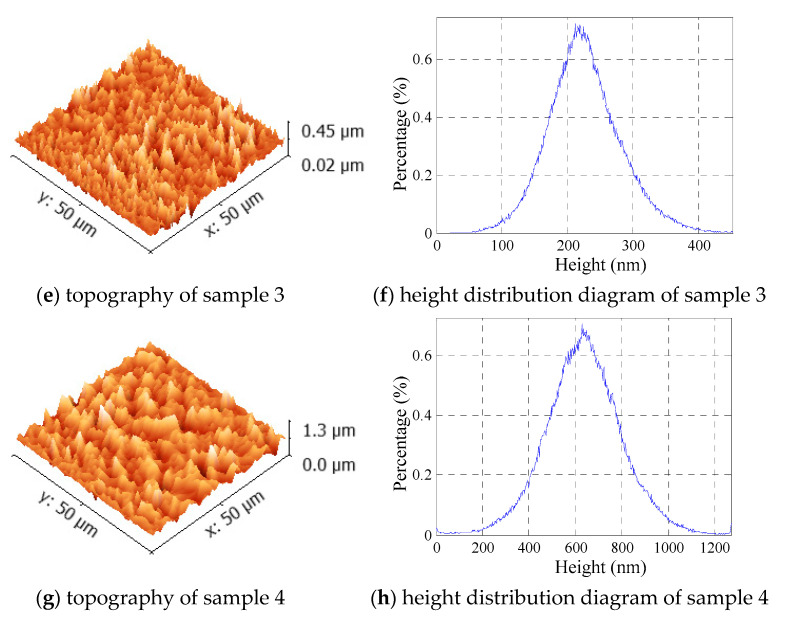
Morphology and height distribution diagram of each sample.

The sampling area of each sample is 2500 µm^2^, and the maximum heights of the roughness peaks of samples 1–4 are approximately 0.07 µm, 0.22 µm, 0.45 µm, and 1.3 µm, respectively, presenting a gradual increase. The skewness value of sample 1 is approximately zero, and the kurtosis value is 3.14, which is close to 3; Samples 2 and 3 are positively skewed, and their kurtosis values are more than 3; the skewness value of sample 4 is approximately zero, and its kurtosis mean value is 3.86. The first scanning analysis results are shown in Figure 6. The height distribution diagrams of samples 1–4 are broadly bell-shaped. Overall, the rough peak height distribution of each sample is approximately normal. 

**Figure 7:**
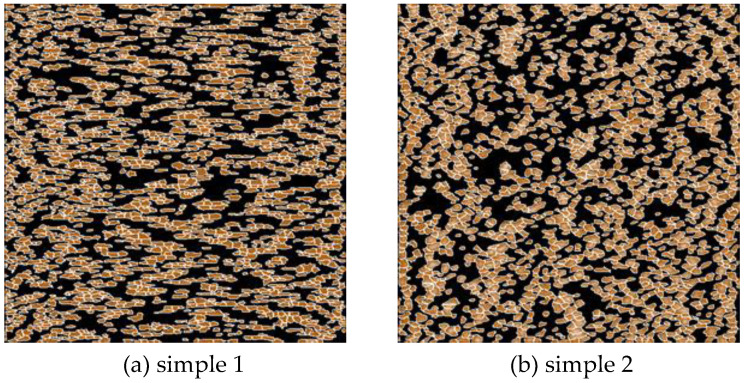

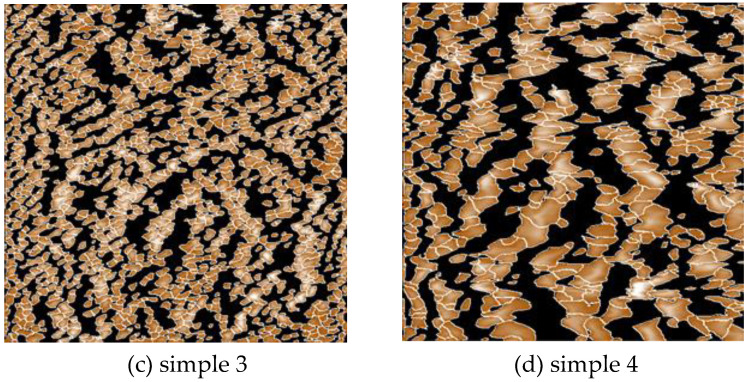
Grain size analysis. (**a**) sample 1, (**b**) sample 2, (**c**) sample 3, (**d**) sample 4.

For determining the peak density and size, the particle size analysis function of NanoScope_Analysis was conducted for the scanning images of samples 1–4, as shown in Figure 7. The mean peak density values, η, and mean asperity tip radius, ρ, are listed in Table 5. The particle distributions and particle sizes of samples 1–3 are more uniform, whereas the uniformity of the particle distribution and particle size of sample 4 is not well.

### 4.2. Friction Experimental Results

The plasticity index, Ψ, suggested by Greenwood and Williamson [9] is the most important parameter to be considered in the analyses of contact rough surfaces. It has the form:

(36) $$\Psi =\frac{2E}{\pi KH}\sqrt{\frac{\sigma_{s}}{\rho }}$$

where *E* is the Hertz elastic modulus, *K* is the hardness factor, *K* = 0.454 + 0.41*v*, *H* is the hardness of the softer material, and $\sigma_{s}$ is the standard deviation of the asperity heights.

The height distribution of each sample is similar to a Gaussian distribution. In this study, the distributions of the four samples are treated as Gaussian distributions. The sample material is 100 monocrystalline silicon: *H* = 11.5 GPa, *E* = 169 GPa, *v* = 0.18, *a*_0_ = 0.542 nm, and *l_s_* = 0.96.

**Figure 8:**
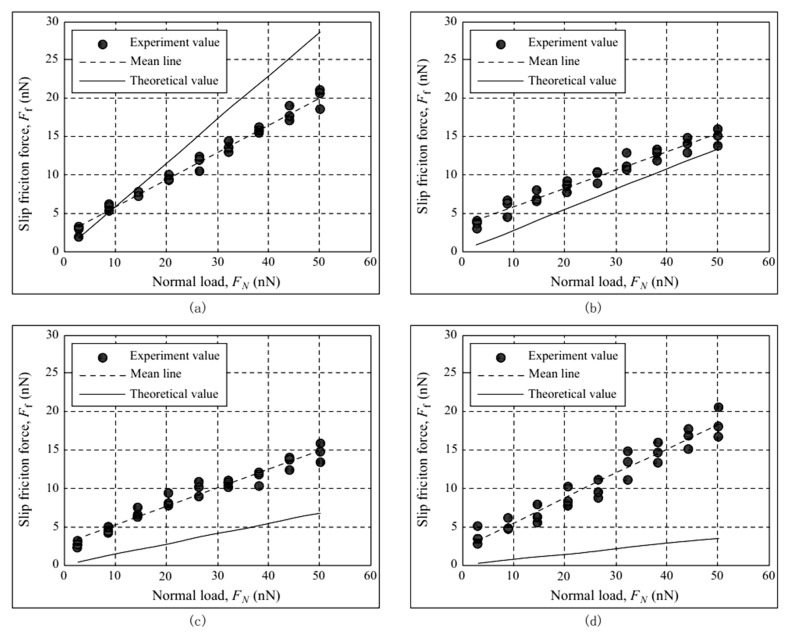
Dry friction experimental results with AFM: (**a**) sample 1, (**b**) sample 2, (**c**) sample 3, (**d**) sample 4.

The friction test results of the four samples are shown in Figure 8. The friction test results of sample 1 are shown in Figure 8a. Its plasticity index is 0.4553 and surface roughness Sa = 8.9 nm; its roughness is the smallest among the four samples. The friction of sample 1 increases approximately linearly with the increase in the normal force, which is consistent with the Amontons friction law [1]. When the normal force is small, the theoretical calculated value of friction obtained using Equation (25) is in good agreement with the experimental value. However, with the gradual increase in the normal load, the difference between the theoretical calculated and experimental values also increases.

The friction test results of sample 2 are shown in Figure 8b. For sample 2, Ψ=0.9551 and Sa = 18.8 nm, and it is coarser than sample 1. The experimental value of friction is lower than that of sample 1, which is related to the factors of the roughness peak density and average roughness peak radius of sample 2 being smaller than those of sample 1. With the increase in the plasticity index, the theoretical calculation value of friction is significantly reduced compared to sample 1, and the experimental value is also reduced; however, the theoretical calculation value is slightly greater than the experimental result.

The friction test results of sample 3 are shown in Figure 8c; its Sa = 43.1 nm and Ψ=1.8348, and it is coarser than sample 2. However, there is no significant difference in the experimental values of samples 3 and 2. The experimental values of sample 3 are smaller than the theoretical values; concurrently, the theoretical values of sample 3 become greater than that of sample 2 with the increase in the plasticity index.

The friction test results of sample 4 are shown in Figure 8d, and its Sa = 125 nm and Ψ=3.8347, making it the coarsest of the four samples, whereas its experimental value of friction does not reduce with the increase in the roughness. For sample 4, the ball radius of the probe is not sufficiently long, which is significantly affected by the height of the rough peak and the gap between the rough peaks, and the mechanical component of friction is also increased. The experimental values of sample 4 are the most divergent, whereas its theoretical values of friction are the smallest, and the difference between the theoretical and experimental values is the largest among all samples.

**Figure 9:**
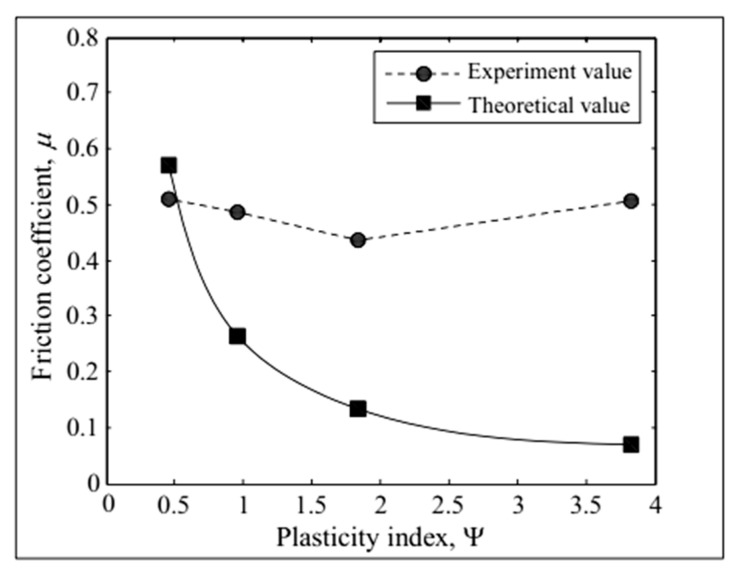
Friction coefficient versus plasticity index.

The relationships between the experimental and theoretical values of the friction coefficient and the plasticity index are shown in Figure 9. The experimental friction coefficient in the figure is the average value of the ratio of the friction force and normal force at each sampling. For samples 1–4, the plasticity index increases gradually. The theoretical friction coefficient decreases gradually with the increase in the plasticity index, which is positively correlated with the roughness. For samples 1–3, the experimental values of the friction coefficient decrease with the increase in the plasticity index, whereas for sample 4, the friction coefficient first decreases and then increases up to the largest plasticity index. It can be seen from Figure 9 that a small surface roughness is associated with a small error between the experimental and theoretical values of the friction coefficient. However, friction is an extremely complex process.

## 5. Conclusions

The novelty of this work was to deal with the slip friction calculation of rough surfaces contact with the potential energy theory and carry out the experimental verification with the contact angle measuring instrument and AFM. In this study, monocrystalline silicon samples with different roughness surfaces were prepared by wet etching, and the surface free energies of the samples were obtained using a contact angle measuring instrument. The morphology parameters of each sample were measured by AFM, and spherical-rough flat contact friction experiments were conducted. Finally, the experimental results were analyzed and compared with the theoretical calculated values. The comparison results showed that for the samples with a small plasticity index, the deviation of the theoretical calculated and experimental values of the friction force and the coefficient was small. For the samples with a large plasticity index, the corresponding deviation was large. In general, for a small plasticity index (Ψ≤1), the predicted values of the friction force and friction coefficient by this model presented a relatively acceptable deviation range from the experimental values, which can meet certain engineering requirements in MEMS.

However, the unavoidable impact factors in the actual friction process, such as the interaction among the asperities [35,36], abrasive wear, and so on [37], are ignored by assumptions in this model, so this model is more suitable for the friction situations of low load, small plasticity index, dry environment, etc.

In future studies, ball tip probes with different spherical radii will be used in dry slip friction experiments. Moreover, by comparison, the influence laws of different spherical radii on the calculation accuracy of the model presented in this paper will be determined, and the model will be further improved.

## Figures and Tables

**Table 1:** List of functions of Poisson’s ratio [29].

Symbol	Function
$C_{v}$	1.234+1.256v
α	0.25+0.125v
β	0.174+0.08v

**Table 2:** Surface tension values of measuring liquids (mJ/m^2^) [34].

Measuring Liquid	$\gamma_{L}$	$\gamma_{L}^{d}$	$\gamma_{L}^{p}$
Distilled Water	72.8	21.8	51.0
Diiodomethane	50.8	48.5	2.3

**Table 3:** Contact angles and surface energies of samples (monocrystalline silicon wafer).

No	Distilled Water (°)	Diiodomethane (°)	$\gamma_{S}^{d}\;({\mathrm{mJm}}^{-2})$	$\gamma_{S}^{p}\;({\mathrm{mJm}}^{-2})$	$\gamma_{S}\;({\mathrm{mJm}}^{-2})$
Sample 1	41.9 ± 2.8	36.5 ± 3.3	37.43	27.23	64.66
Sample 2	39.4 ± 2.2	32.5 ± 1.4	39.01	27.95	66.96
Sample 3	45.0 ± 3.5	35.6 ± 4.2	37.80	25.53	63.35
Sample 4	43.6 ± 1.9	32.7 ± 2.7	38.96	25.88	64.84

**Table 4:** Physical parameters of the silicon spherical probe used in this work.

Name	*R* (µm)	*w* (µm)	*b* (µm)	*l* (µm)	*h* (µm)	*E* (N/m^2^)	*G* (N/m^2^)	*k_n_* (Nm^−1^)	*k_l_* (Nm^−1^)
silicon probe	9.55	40.05	3.51	240.20	54.40	1.69 × 10^11^	0.5 × 10^11^	5.28	40.61

**Table 5:** Roughness parameters of samples.

No.	Etching Time	Sa (nm)	Sq (nm)	Ssk	Sku	*η* (nm^−2^)	ρ (nm)	Ψ
Sample 1	5 min	8.9 ± 0.6	11.4 ± 0.5	0.048 ± 0.008	3.14 ± 0.03	7.604 × 10^−7^	4.614	0.4553
Sample 2	10 min	18.8 ± 1.3	24.1 ± 0.8	0.316 ± 0.037	3.75 ± 0.17	7.488 × 10^−7^	2.216	0.9551
Sample 3	15 min	43.1 ± 2.8	55.6 ± 1.9	0.205 ± 0.103	3.49 ± 0.07	5.192 × 10^−7^	1.385	1.8348
Sample 4	20 min	125 ± 4.3	168 ± 8.1	−0.046 ± 0.015	3.26 ± 0.62	1.224 × 10^−7^	0.958	3.8347

## Nomenclature

$a_{0}$	lattice constant
$A_{0}$	real contact area
$F_{\mathrm{slip}}$	sliding friction force
$F_{N}$	external normal force
g(T)	temperature coefficient
h	separation based on surface heights
k	position commensurate coefficient
K	hardness factor, 0.454+0.41v
P	external normal force
R	radius of the sphere
z	height of an asperity measured from the mean of the asperity heights
β	surface roughness parameter, ηρσ
γ	energy of adhesion
ΔU	interfacial potential difference
w	adhesion energy of the contact interface
ϕ	distribution function of the asperity heights
η	area density of the asperities
ρ	asperity tip radius of curvature
μ	static friction coefficient
v	Poisson’s ratio of the softer material
σ	standard deviation of the surface heights
$\sigma_{s}$	standard deviation of the asperity heights
ω	interference
$\omega_{c}$	critical interference at the inception of plastic deformation
Ψ	plasticity index
